# Rethinking the TNM Classification Regarding Direct Lymph Node Invasion in Pancreatic Ductal Adenocarcinoma

**DOI:** 10.3390/cancers14010201

**Published:** 2021-12-31

**Authors:** Fiona Speichinger, Mihnea P. Dragomir, Simon Schallenberg, Florian N. Loch, Claudius E. Degro, Ann-Kathrin Baukloh, Lisa Hartmann, Ioannis Pozios, Christian Schineis, Georgios Antonios Margonis, Johannes C. Lauscher, Katharina Beyer, Carsten Kamphues

**Affiliations:** 1 Charité–Universitätsmedizin Berlin, Corporate Member of Freie Universität Berlin and Humboldt–Universität zu Berlin, Department of General and Visceral Surgery, Hindenburgdamm 30, 12203 Berlin, Germany; florian.loch@charite.de (F.N.L.); claudius-erwin.degro@charite.de (C.E.D.); ann-kathrin.baukloh@charite.de (A.-K.B.); lisa.hartmann@charite.de (L.H.); ioannis.pozios@charite.de (I.P.); christian.schineis@charite.de (C.S.); johannes.lauscher@charite.de (J.C.L.); katharina.beyer2@charite.de (K.B.); carsten.kamphues@charite.de (C.K.); 2 Charité-Universitätsmedizin Berlin, Freie Universität Berlin, Humboldt-Universität zu Berlin and Berlin Institute of Health, Institute of Pathology, 10117 Berlin, Germany; mihnea.dragomir@charite.de (M.P.D.); simon.schasllenberg@charite.de (S.S.); 3 German Cancer Consortium (DKTK), Partner Site Berlin, German Cancer Research Center (DKFZ), 69210 Heidelberg, Germany; 4 Berlin Institute of Health, Anna-Louisa-Karsch-Straße 2, 10178 Berlin, Germany; 5 Department of Surgery, The Johns Hopkins University School of Medicine, 600 N. Wolfe Street, Blalock 688, Baltimore, MD 21287, USA; margonig@mskcc.org

**Keywords:** pancreatic ductal adenocarcinoma, direct lymph node invasion, TNM classification

## Abstract

**Simple Summary:**

Due to the rising burden of pancreatic cancer and poor outcomes, a precise, post-operative cancer staging for further and individualized therapy is needed. In the latest cancer classification system, the lymph node invasion mechanism is not addressed. Due to different outcomes regarding the lymph node invasion, we suggest a rethinking of the current system.

**Abstract:**

Mechanisms of lymph node invasion seem to play a prognostic role in pancreatic ductal adenocarcinoma (PDAC) after resection. However, the 8th edition of the TNM classification of the American Joint Committee on Cancer (AJCC) does not consider this. The aim of this study was to analyse the prognostic role of different mechanisms of lymph node invasion on PDAC. One hundred and twenty-two patients with resected PDAC were examined. We distinguished three groups: direct (per continuitatem, Nc) from the main tumour, metastasis (Nm) without any contact to the main tumour, and a mixed mechanism (Ncm). Afterwards, the prognostic power of the different groups was analysed concerning overall survival (OS). In total, 20 patients displayed direct lymph node invasion (Nc = 16.4%), 44 were classed as Nm (36.1%), and 21 were classed as Ncm (17.2%). The difference in OS was not statistically significant between N0 (no lymph node metastasis, *n* = 37) and Nc (*p =* 0.134), while Nm had worse OS than N0 (*p <* 0.001). Direct invasion alone had no statistically significant effect on OS (*p =* 0.885). Redefining the N0 stage by including Nc patients showed a more precise OS prediction among N stages (*p =* 0.001 vs. *p =* 0.002). Nc was more similar to N0 than to Nm; hence, we suggest a rethinking of TNM classification based on the mechanisms of lymph node metastases in PDAC. Overall, this novel classification is more precise.

## 1. Introduction

According to the International Agency for Research on Cancer, more than 495,000 new cases of pancreatic cancer were recorded worldwide in 2020. Moreover, pancreatic cancer is the seventh most frequent cause of cancer-related deaths [1,2]. The prognosis of patients suffering from pancreatic ductal adenocarcinoma (PDAC) is very poor, with high lethality rates, leading to a five-year survival rate of less than 10% [1,2]. The most powerful predictor of survival after surgery is lymph node status [3,4,5,6]. Previous studies [7,8,9,10,11,12] have analysed the mechanisms of lymph node invasion and compared the prognosis of direct lymph nodes from the main tumour through cancer related angiogenesis and immunosuppression via crosstalk between the cancer endothelium and the surrounding microenvironment and lymph node metastasis without any contact with the main tumour [13,14]. These small studies showed that the mechanisms underlying lymph node invasion may have effects on the survival rate, however, the data are contradictory [7,8,9,10,11,12]. In addition, the latest TNM classification—the 8th edition of the American Joint Committee on Cancer (AJCC) with changes to the T and N categories—does not consider the mechanisms of lymph node involvement [15,16,17,18,19].

Our study provides data that support a redefinition of the N stage by including the mechanism of lymph node involvement [12]. We believe that this could lead to a more precise description of lymph node status, providing an individualized prognosis of PDAC patients compared with the current TNM classification.

## 2. Materials and Methods

### 2.1. Patients and Clinical Data

A total of 136 patients who underwent pancreatic resection at the Department of General and Visceral Surgery at the Charité University Hospital, Campus Benjamin Franklin, Berlin, Germany, between 2008 and 2021, were retrospectively analyzed. Thirteen patients with 30-day mortality (9.6% overall postoperative mortality) and one patient with carcinoma of the duodenum were excluded. Overall, 122 patients suffering from PDAC were included in this study. All patients underwent pancreatic surgery (pylorus-preserving pancreaticoduodenectomy = PPPD/Whipple procedure or left-sided pancreatic resection) according to the current guidelines, after the indication for surgery and chemotherapy was confirmed by an interdisciplinary tumour board. Clinicopathological characteristics such as age, sex, follow-up, and recurrence-free survival were collected for each patient (Table 1). The study was approved by the Ethics Committee of the Charité University Medical Department in Berlin (EA4/020/19).

### 2.2. Histopathological Assessment: Grossing, Histological Examination and Reexamination of Lymph Node Metastases

Resected specimens were fixed in 10% buffered formalin before grossing. After overnight fixation, the specimens were stained before preparation (anterior margin yellow, medial margin blue, posterior margin black, bile duct green and pancreas parenchyma red). As a first step, resection margins of oral and aboral duodenum, biliary duct and pancreas parenchyma were identified and were completely embedded. As a second step, the axial method was used, slicing the specimen from apical to caudal in 5-mm-thick slices. Next, the tumour was detected, described and measured, including the minimum distance to all relevant anatomical structures and the previously stained circumferential soft tissue margins. We next embedded the tumour in closest relation to the ampulla, pancreatic duct, duodenum, bile duct, pancreas parenchyma resection margin and to the anterior, posterior, medial circumferential soft tissue margins. 

Regarding lymph node grossing, according to our protocol, we embedded all macroscopic detectable lymph nodes, minimally 12, and if this number was not achieved, we embedded the peripancreatic fat completely. If the surgeons submitted other regional lymph nodes separately, these were completely sampled. On average, per case, we embedded 18 blocks.

Histological examination was done according to the 8th edition of the TNM classification (AJCC) and included: defining the cancer subtype, grading, pTNM-classification, vascular, lymphatic, and perineural invasion, detection of precursor lesions and R-status analysis. R-status, at our institution, is defined as direct invasion of the tumour in the resection margins/circumferential soft tissue margins (0 mm).

**Figure 1:**
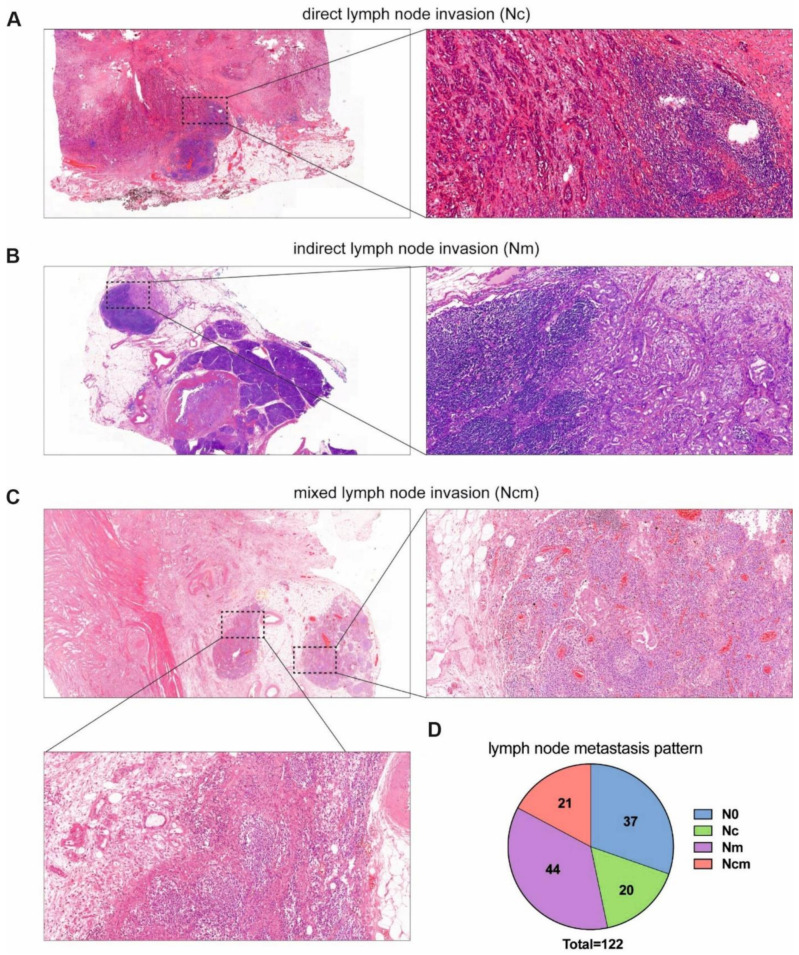
Histological sections with haematoxylin–eosin staining (at 20x magnification and the zoom-in cassettes at 100x magnification) of the different types of lymph node invasion in pancreatic ductal adenocarcinoma: (**A**) direct lymph node invasion by the main tumour per continuitatem, Nc; (**B**) indirect lymph node invasion without any contact to the main tumour, Nm; (**C**) Mixed lymph node invasion, Ncm; (**D**) pattern of the different lymph node types in our study.

Two pathologists independently re-evaluated the histology slides for the lymph node metastases reclassification. Lymph nodes were categorized according to the mechanism/presence of invasion: direct (Nc), metastasis (Nm), mixed mechanism (Ncm), and node-negative (N0). Nc was defined as direct lymph node invasion by the tumour (Figure 1A), Nm was defined as regional lymph node metastasis without any contact to the tumour (Figure 1B) as previously described [7,8,9,10,11,12], and Ncm was defined as a combination of both (Figure 1C). If discrepancies existed between the two pathologists, these were resolved by extensively discussing the case and if no consensus was met, the opinion of a third pathologist was asked. 

### 2.3. Statistical Analysis

For the statistical analyses, we used SPSS version 27.0 (IBM). Overall (OS) were plotted as Kaplan–Meier curves and survival differences were calculated using the log-rank test. Clinicopathologic characteristics were compared using the Pearson–chi-quadrant test. Significant univariate variables (*p* < 0.10) in the Cox proportional hazards regression model were proofed of proportional hazards assumption and further analysed in the multivariate model. For comparison of the results from the other groups, we analysed a weighted median of OS. A *p*-value of <0.05 was considered as a statistically significant difference. Graphics were designed using CorelDRAW® Graphic Suite 2021 (Corel Corporation, Ottawa, ON, Canada). 

## 3. Results

### 3.1. Demographics 

Table 1 shows the clinicopathological characteristics for the different lymph node invasion types of the 122 patients with PDAC after radical resection. In total, 56 patients (45.9%) were female. The median age of the patients was 70.3 (35.4–86.7) years. In 101 (82.8%) patients, the tumour was located mainly in the pancreatic head, and 96 (78.7%) of the resections were R0. According to the 8th Edition of the TNM classification, 57 tumours (46.7%) were stage T3, followed by 56 tumours at T2 (45.9%), 9 tumours at T1 (7.4%), and no tumours at T4; further, 37 (30.3%) patients were node-negative (N0), 54 (44.3%) were N1, and 31 (25.4%) were N2, while 51 (41.8%) patients had angiolymphatic invasion, 25 (20.5%) had venous invasion, and 78 (63.9%) had perineural invasion. According to the involved lymph node mechanisms, we found 20 (16.4%) patients with direct lymph node invasion (Nc), 44 (36.1%) patients with lymph node metastasis without any contact with the tumour (Nm), and 21 (17.2%) patients showing mixed lymph node invasion (Ncm) (Figure 1). The median number of analysed lymph nodes per patient was 15 (range 3–50) for all groups, 11 (range 3–50) for N0 patients, 12 (range 4–46) for Nc, 15 (range7–43) for Nm, and 20 (range 8–43) for patients with a mixed lymph node invasion. Statistically, more lymph nodes were analysed in the Ncm group compared with the N0 group (*p* = 0.035) as well as in the Nc group (*p* = 0.04). Interestingly no statistically significant difference was seen in the lymph node Ratio (LNR) between the groups (Table 2). In total, we analysed 2079 lymph nodes. In total, 334 lymph nodes were positive, and direct invasion was found in 73 lymph nodes (21.9%)—on average, 1–5 lymph nodes with direct invasion per patient. Statistically significant difference between the groups were only seen in the N stage (*p* < 0.001), tumour location (*p* = 0.035), and angiolymphatic invasion (*p* < 0.001).

### 3.2. Survival Analysis

The median overall survival of the entire cohort was 21.6 months with a 95% confidence interval (CI) from 14.3 to 28.8 months and the recurrence-free survival was 13 (8.4–17.6, 95% CI) months. The one-year survival of all patients was 61.9%, the three-year survival 31.6%, and the five-year survival was 24.3%. 

#### 3.2.1. Analysis by the Mechanism of Lymph Node Invasion

According to the invasion mechanisms of lymph nodes, the one-year survival of the different groups showed 74.8% in N0, 52.1% in Nc, 61.6% in Nm, and 47.7% in Ncm. The three-year survival was 62.1% in N0, 38% in Nc, 12.8% in Nm, and no patient lived after three years in the Ncm group. The five-year survival of the different groups showed 50.2% in N0, 38% in Nc, and no patient survived five years or more in the Nm and Ncm groups. 

**Figure 2:**
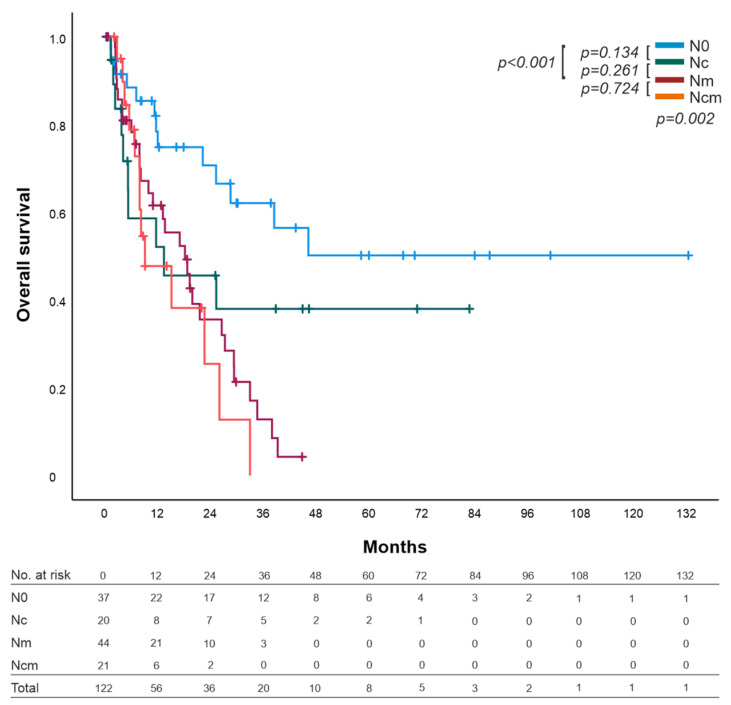
Overall survival (OS) of patients with PDAC distinguished by node-negative (N0), per continuitatem (Nc), lymph node metastasis (Nm), and combination of per continuitatem and lymph node metastasis (Ncm). Overall statistical significance difference was *p* = 0.002; no statistical significance difference between N0 and Nc (*p =* 0.134); a significant statistically difference between N0 and Nm was found (*p* ≤ 0.001); Nc and Nm showed no statistically significant difference, but their curves diverged strongly (*p =* 0.261). No statistically significant difference between Nm and Ncm was found (*p* = 0.724).

The median OS of the N0 group could not be calculated since less than 50% of the patients in the N0 group died. The median OS of the other groups was 13.5 (0–37.1, 95% CI) months for Nc, 18.2 (11–25.5, 95% CI) months for Nm patients, and 9.2 (1.4–16,9, 95% CI) months for the Ncm group (Figure 2).

Overall comparison of OS by the mechanisms of lymph node invasion indicated a statistical significance (*p* = 0.002). Paired comparisons of the two groups showed no statistically significant difference between N0 and Nc (13.5 months; *p* = 0.134), however, a statistically significant difference between N0 and Nm (18.2 months; *p* < 0.001), as well as between N0 and Ncm (9.2 months; *p* = 0.01) was found. Nc showed no statistically significant difference with Ncm (13.5 vs. 9.2 months; *p* = 0.458) or Nm (13.5 vs. 18.2 months; *p* = 0.261), but their curves diverged strongly. No statistically significant difference was found between Nm and Ncm (*p* = 0.724) (Figure 2).

#### 3.2.2. Overall Survival by TNM Classification and UICC Stages 

**Figure 3:**
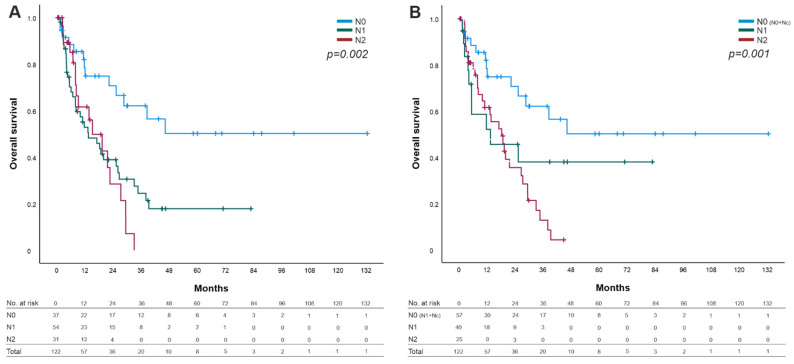
Overall survival (OS) sorted by N categories. (**A**) OS of the 8th edition of the AJCC Cancer Staging Manual for PDAC. (**B**) Revised N categories (N0-R = N0 + Nc) with statistically significant difference compared with the actual N categories (*p*= 0.002 vs. *p*= 0.001).

As we determined no impact on survival by direct lymph node invasion alone (Table 2), we defined a reviewed N stage by combining the N0 group with the Nc group as a reviewed N0 stage (N0-R = N0 + Nc; Figure 3B). The overall comparison in our reviewed N stage showed greater statistically significant difference (*p* = 0.001) in contrast to the current N stage of the 8th edition of the AJCC (*p* = 0.002; Figure 3A) concerning OS. 

The same observations were identified for the UICC stages between the current system and the reviewed stages (*p* = 0.009 vs. *p* = 0.008). As described above, we combined the group of direct lymph node invasion (Nc) with the node-negative group. (data not shown).

### 3.3. Prognosis Factors

Resection margin status venous, and angiolymphatic invasion, as well as our revised N stage (N0-R = N0 + Nc/Nm + Ncm) showed a statistically significant difference in the overall survival in the univariate analysis using the Cox proportional hazards regression model and seemed to be predictors of shorter OS (Table 2). To determine the lymph node ratio (LNR) we build three groups with different cut offs (1: >0 and <0.2; 2: ≥0.2 and <0.4; 3: ≥0.4), as described before [20,21,22] with no statistically significant difference. After proofing the proportional hazards assumption, further analysis of these variables in the multivariate analysis indicated venous invasion (VI) as well N0-R as predictors for overall survival. Direct node invasion (Nc) alone is not a prognostic factor of worse survival (*p* = 0.885).

### 3.4. Contribution of Disease Recurrence by Node Invasion Mechanism 

Overall, 71 (58.2%) patients suffered a disease recurrence. Thereof, 19 (26.8%) patients were in the N0 group, 8 (11.3%) were in the Nc, 29 (40.8%) were in the Nm, and 15 (21.1%) patients were in the Ncm group. No statistical differences were detected in disease recurrence patients compared with patients without disease recurrence (*p* = 0.164). In total, we counted 20 (28.2%) patients with a local disease recurrence, 37 (52.1%) with a systemic disease recurrence and 14 (19.7%) patients with a combination of both. No differences were seen between the groups based on localization (*p* = 0.118).

Direct invasion (Nc + Ncm vs. N0 + Nm) alone had no effect on disease recurrence (*p* = 1.0). No differences were seen between N0 and Nc (*p* = 0.562) nor between Nc and Nm (*p* = 0.089).

## 4. Discussion

Since lymph node status is the strongest predictor of survival in PDAC [3,4,5,6], a more detailed classification of the N stage is needed regarding the different mechanisms of lymph node invasion for an individualized therapy after resection. No distinction is made among the local proliferation of the tumour by infiltrating lymph nodes externally per continuitatem through angiogenesis and immunosuppression [13,14] and “real” lymphogenic spread in the current TNM classification system—despite the assumption that a local proliferation differs from a systemic proliferation by the lymphatic system. 

Some groups have attempted to find the prognostic factors via lymph node mechanisms but have shown some contradictory data [7,8,9,10,11,12]. 

Thus, we discuss our data here in order to rethink TNM classification. We observed a similar patient cohort in our study concerning clinicopathologic characteristics to the other groups [7,8,9,10,11,12]. 

Regarding the proportions of the different lymph node types, we detected most patients with directly involved lymph nodes by the tumour (16.4% Nc) compared with the others (3.6–14.3%), as well as mixed node invasion (17.2% vs. 1.5–12.6%) (Table 3). Hoshikawa et al. [11] performed an analysis with all lymph nodes together and showed that 27.6% of lymph nodes invaded per continuitatem. In the same way, we detected almost the same (21.9%) from our analysis. Node-negative patients occur in equal amounts over all groups, except in the study by Hoshikawa et al. [11], who counted only 10 node-negative patients (10.2%). Only 36.1% of our patients showed real lymph node metastasis (Nm), and this was the lowest proportion detected in the current literature. Reversed ratios of Nc/Nm may indicate a more precise analysis of the lymph nodes in PDAC compared with former studies.

Regarding the number of involved direct lymph nodes per patient, we counted 1–5 lymph nodes—similar to Byun et al. [12]. Other groups counted 1–2 [7,8] or 1–7 [10,11], and a maximum mean of 2.4 lymph nodes per continuitatem were detected in the study by Buc et al. [9]. 

Discrepancies in the proportions of the different types may occur due to the analysis and variable time period of the recruited patients. The first three studies [7,8,9] analysed patients between 1990–2009, due to rapid changes in medicine—which is a probable explanation. Furthermore, all histological sections were analysed by two different pathologists in the current study as well as in the latest studies [10,11,12]. Unlike in the initial studies, they did not review the sections [7,9] or, at least, not all of them [8].

Since Konstantinidis et al. and Pai et al. found only one–two invaded direct lymph nodes, they excluded all patients with more than two lymph node metastases [7] or excluded patients from a further review with three or more lymph node metastases [8]. The proportions of the Nc group are likely underestimated. 

Despite the graphs diverging greatly (Figure 2) of the Nc and Nm/Ncm groups in contrast to the similar parallel curves of N0 and Nc (Figure 2) we detected no statistically significant difference of OS in the Nc group compared with Nm or Ncm. The missing statistically significant difference is probably attributed by the small group of patients supported by the worse statistically significant difference in the three- and five-year survival of the Nm and Ncm groups. Similar observations were seen in other groups [8,10,12], supporting the hypothesis that Nc might be a different entity compared with Nm and is more related to N0 (Table 4).

Pai et al. (2012) [8] analysed 308 patients and found no statistically significant difference between N0 and Nc in overall survival (*p =* 0.609) but discovered improved overall survival in Nc compared with Nm (*p =* 0.001). This result is reflected by the comparison of the five-year survival (N0 31%, Nc 36%, and Nm 8%) [8]. Williams et al. (2015) showed similar overall survival between Nc and N0 (*p =* 0.719), statistically significant differences between N0 and Nm (*p <* 0.001), however, no statistically significant difference between Nc and Nm (*p =* 0.190) was observed [10]. Supporting our results, in 2019, Hoshikawa et al. analysed 98 patients and showed similar survival between the Nc and the N0 group [11]. 

According to our knowledge, this study underlines the latest results in this field by Byun et al. (2021), who had also proposed a change of the N stage regarding the lymph node invasion per continuitatem. Moreover, they did not observe any statistically significant difference in the disease-free survival (DFS) of N0 and Nc (*p =* 0.999) but statistically significant difference between Nc and Nm (*p =* 0.002) as well as between Nc and Ncm (*p* < 0.001). Furthermore, they showed higher statistically significant differences in the disease-free survival of a revised TNM classification compared with the current 8th edition of the AJCC classification system (*p =* 0.003, revised *p <* 0.001) similar to our data (*p =* 0.002, N0-R (N0 + Nc)*p =* 0.001). Comparing this study with all the other studies is limited, since they analysed the disease-free survival (DFS) and showed no data regarding the overall survival [12]. 

In contrast, Konstantinidis et al. (2010) [7] detected a similar unfavourable overall survival of Nc and Nm patients compared with N0 patients (*p =* 0.67). However, they excluded all patients with more than two detected positive lymph nodes and the mixed type Ncm [7]. Therefore, the results are limited by this selection and do not illustrate the collective of PDAC patients, evidenced by the fact of the significantly lower five-year survival of all patients (*n* = 517) compared with the other groups inclusive of our data analysis (17.3% vs. 24.3–32%) [8,9,10]. 

Likewise, Buc et al. showed an unfavourable survival in patients with direct invasion compared with the node-negative group (*p* = 0.037). No statistically significant difference was seen between Nc and Nm (*p =* 0.57). However, statistically significant differences between Nm and N0 were found (*p <* 0.001). Interestingly, direct invasion on its own showed no impact on overall survival (*p =* 0.27), which is similar to our data (*p =* 0.220) [9]. 

The detected prognostic factors of survival by the different groups included margin status after resection, T stage, grade, angiolymphatic and venous invasion, and involved lymph nodes, in general, related to the current data (Table 2). Direct lymph node invasion (Nc) was not an independent prognostic factor [9,11,12] as confirmed by our observations (*p* = 0.885).

Interestingly, no statistical difference was discovered in the appearance of disease recurrence between the lymph node types as well as the location (local/systemic) of the tumour recurrence. Byun et al. published the same results in 2021 [12]. This owes to the small patient cohort size and, thus, requires further investigations in larger-scale studies. 

The key strengths of our research are the histological analysis by two independent pathologists considering the 8th edition of the TNM classification of the American Joint Committee on Cancer (AJCC). The comparison of all above-mentioned studies showed that a wide range of lymph nodes invaded per continuitatem were detected [7,8,9,10,11,12]. Despite the smaller group of patients, we observed similar contributions and had, in fact, proportionally more patients with direct invasion, compared with the other studies [7,8,9,10,11,12], revealing the importance of precisely analysing the correct classification of each patient [23]. Our study supports the latest published results [12] and is, according to our knowledge, the only paper that has, so far, considered the 8th AJCC system in Europe with the highest proportion of lymph nodes per continuitatem.

Limitations, such as the small number of patients, retrospective analyses, local sampling protocols, statistically significant difference in the number of lymph nodes in the different groups, and single-institution analysis require further and larger studies with international collaborations to overcome. Especially, a statistically powered study is necessary to validate our hypothesis of rethinking the N classification. Due to cancer-related angiogenesis and immunosuppression through interactions of the cancer endothelium with immune cells, further focused studies for an individualized anti-angiogenesis and immunomodulatory therapy are urgently required [13,14].

Another limitation is that we did not find any patient in the Nc group classified N2 regarding the latest TNM classification system (≥4 lymph node metastasis); however, we categorized 14 patients of the Ncm group N2. Byun et al. found 1 Nc patient and 33 Ncm patients [12]. Either PDAC has only a limited tendency of invasion of the tumour surrounding lymph nodes per continuitatem—supporting the hypothesis that Nc is similar to N0—or we underestimated the direct lymph nodes per patient due to the small groups. 

All of the above results—in accordance with the latest studies—suggest that lymph node invasion per continuitatem is a different entity despite the normal N classification. Moreover, Nc seemed to be more similar to N0 lymph nodes. For a more precise prognosis of PDAC patients, we need to reconsider the TNM classification regarding the N stage.

## 5. Conclusions

In conclusion, we found similar characteristics in patients with direct lymph node invasion compared with node-negative patients after PDAC resection; hence, we suggest a redefinition of the TNM classification based on the mechanism of lymph node metastases in patients with PDAC. Overall, this novel classification has a more precise prognostic power. 

## Figures and Tables

**Table 1:** Clinicopathologics arranged by the different lymph node types.

		N0 (*n* = 37)	Nc (*n* = 20)	Nm (*n* = 44)	Ncm (*n* = 21)	Total (*n* = 122)	*p*-Value
Median Age (range)		70.3 (35.4–84.4)	70.3 (52.6–83)	71.6 (36.8–84.5)	69.2 (41.8–86.7)	70.3 (35.4–86.7)	0.402
Gender							0.459
	female	20	10	19	7	56 (45.9)	
	male	17	10	25	14	66 (54.1)	
T stage							0.693
	T1	4	1	2	2	9 (7.4)	
	T2	13	10	24	9	56 (45.9)	
	T3	20	9	18	10	57 (46.7)	
	T4	0	0	0	0	0	
N stage							<0.001
	N0	37	0	0	0	37 (30.3)	
	N1	0	20	27	7	54 (44.3)	
	N2	0	0	17	14	31 (25.4)	
Resection							0.340
	R0	29	18	35	14	96 (78.7)	
	R1	8	2	9	7	26 (21.3)	
Grade							0.090
	G1	5	0	0	0	5 (4.1)	
	G2	20	11	21	11	63 (51.6)	
	G3	12	9	22	10	53 (43.4)	
	G4	0	0	1	0	1 (0.8)	
Location							0.035
	head	24	17	41	19	101 (82.8)	
	corpus	3	1	0	1	5 (4.1)	
	tail	10	2	3	1	16 (13.1)	
Invasion							
	ALI	1	8	28	14	51 (41.8)	<0.001
	VNI	3	6	9	7	25 (20.5)	0.115
	PNI	17	14	31	16	78 (63.9)	0.486

N0: node negative; Nc: direct node invasion (per continuitatem); Nm: regional lymph node metastasis; Ncm: mixed node invasion; ALI: angiolymphatic invasion; VNI: venous invasion; PNI: perineural invasion. Percentage in brackets.

**Table 2:** Univariate and multivariate analysis of prognostic factors.

	Univariate	Multivariate				
	HR	CI 95%	*p*-Value	HR	CI 95%	*p*–Value
age (<65/>65 years)	01.217	0.708–2.091	0.478			
sex (male/female)	0.671	0.411–1.097	0.112			
T stage (T1/T3)	1.539	0.645–3.674	0.331			
resection (R0/R1)	2.706	1.507–4.859	0.001	1.627	0.838–3.160	0.151
grade (G2/G3)	1.481	0.901–2.435	0.121			
PNI (no/yes)	2.250	0.891–5.683	0.86			
VI (no/yes)	2.387	1.336–4.266	0.003	2.504	1.384–4.515	0.002
ALI (no/yes)	2.378	1.420–3.983	0.001	1.6	0.861–2.973	0.137
LNR (>0-<0.2/≥0.4)	2.138	0.910–5.024	0.081			
Mechanism of lymph node invasion						
Nc (no/yes)	0.952	0.484–1.869	0.885			
N0-R(N0 + Nc)/Nm + Ncm	2.567	1.511–4.359	<0.001	3.024	1.709–5.352	<0.001

HR: hazard ratio; CI: confidence interval; ALI: angiolymphatic invasion; VNI: venous invasion; PNI: perineural invasion; N0: node negative; Nc: direct node invasion (per continuitatem); Nm: regional lymph node metastasis; Ncm: mixed node invasion.

**Table 3:** Proportions of lymph node types of the latest studies.

	Total	N0		Nc		Nm		Ncm	
		*n*	%	*n*	%	*n*	%	*n*	%
Konstantinidis et al. 2010	336	168	50.0%	32	9.5%	131	39.0%	5	1.5%
Pai et al. 2011	380	97	25.5%	35	9.2%	239	62.9%	9	2.4%
Buc et al. 2014	301	87	28.9%	19	6.3%	179	59.5%	16	5.3%
Williams et al. 2015	385	146	37.9%	14	3.6%	220	57.1%	5	1.3%
Hoshikawa et al. 2019	98	10	10.2%	14	14.3%	66	67.3%	x	x
Byun et al. 2021	506	176	34.8%	48	9.5%	218	43.1%	64	12.6%
Current study 2021	122	37	30.3%	20	16.4%	44	36.1%	21	17.2%

N0: node negative; Nc: direct node invasion; Nm: regional lymph node metastasis; Ncm: mixed node invasion. Total number of analysed patients; excluded patients are not considered.

**Table 4:** Overall survival of lymph node types of the latest studies.

	N0		Nc		Nm		Ncm	
	*n*	OS Median	*n*	OS Median	*n*	OS Median	*n*	OS Median
Konstantinidis et al. 2010	168	30.8	32	x	131	x	5	x
Pai et al. 2011	97	30	35	21 *	239	15 **	9	15
Buc et al. 2014	87	57	19	34 **	179	33 **	16	22
Williams et al. 2015	146	40.7	14	48.1	220	25.7 **	5	x
Current study 2021	37	x	20	13.5	44	18.2 **	21	9.2 **
Total	535		120		813		56	
Weighted Median OS		30.8		21		25.7		15

Modified table of Williams et al. [10]: N0: node negative; Nc: direct node invasion; Nm: regional lymph node metastasis; Ncm: mixed node invasion; x: not calculated. * Significant difference with Nm. ** Significant difference with N0. The latest study of Byun et al. was excluded as they calculated only disease-free survival, as were the results of Hoshikawa et al. [11], as they built groups of single lymph nodes and distinguished further between isolated tumour cells and between scatter type.

## Data Availability

A dataset of the present study can be requested from the corresponding author with justification.
